# Factors associated with long-term antiretroviral therapy attrition among adolescents in rural Uganda: a retrospective study

**DOI:** 10.7448/IAS.19.5.20841

**Published:** 2016-07-20

**Authors:** Stephen Okoboi, Livingstone Ssali, Aisha I Yansaneh, Celestin Bakanda, Josephine Birungi, Sophie Nantume, Joanne Lyavala Okullu, Alana R Sharp, David M Moore, Samuel Kalibala

**Affiliations:** 1 The AIDS Support Organization (TASO), Kampala, Uganda; 2 Office of HIV/AIDS, Global Health Bureau, United States Agency for International Development (USAID), Arlington, VA, USA; 3 Department of Health Management and Policy, University of Michigan, School of Public Health, Ann Arbor, MI, USA; 4 BC Centre for Excellence in HIV/AIDS, Vancouver, BC, Canada; 5 Faculty of Medicine, University of British Columbia, Vancouver, BC, Canada; 6 HIVCore/Population Council Washington, DC, USA

**Keywords:** adolescents, HIV treatment, antiretroviral therapy, retention, attrition, community-based delivery, Uganda

## Abstract

**Introduction:**

As access to antiretroviral therapy (ART) increases, the success of treatment programmes depends on ensuring high patient retention in HIV care. We examined retention and attrition among adolescents in ART programmes across clinics operated by The AIDS Support Organization (TASO) in Uganda, which has operated both facility- and community-based distribution models of ART delivery since 2004.

**Methods:**

Using a retrospective cohort analysis of patient-level clinical data, we examined attrition and retention in HIV care and factors associated with attrition among HIV-positive adolescents aged 10–19 years who initiated ART at 10 TASO clinics between January 2006 and December 2011. Retention in care was defined as the proportion of adolescents who had had at least one facility visit within the six months prior to 1 June 2013, and attrition was defined as the proportion of adolescents who died, were lost to follow-up, or stopped treatment. Descriptive statistics and Cox proportional hazards regression models were used to determine the levels of retention in HIV care and the factors associated with attrition following ART initiation.

**Results:**

A total of 1228 adolescents began ART between 2006 and 2011, of whom 57% were female. The median duration in HIV care was four years (IQR=3–6 years). A total of 792 (65%) adolescents were retained in care over the five-year period; 36 (3%) had died or transferred out and 400 (32%) were classified as loss to follow-up. Factors associated with attrition included being older (adjusted hazard ratio (AHR)=1.38, 95% confidence interval (CI) 1.02–1.86), having a higher CD4 count (250+ cells/mm^3^) at treatment initiation (AHR=0.49, 95% CI 0.34–0.69) and HIV care site with a higher risk of attrition among adolescents in Gulu (AHR=2.26; 95% CI 1.27–4.02) and Masindi (AHR=3.30, 95% CI 1.87–5.84) and a lower risk of attrition in Jinja (AHR=0.24, 95% CI 0.08–0.70). Having an advanced WHO clinical stage at initiation was not associated with attrition.

**Conclusions:**

We found an overall retention rate of 65%, which is comparable to rates achieved by TASO's adult patients and adolescents in other studies in Africa. Variations in the risk of attrition by TASO treatment site and by clinical and demographic characteristics suggest the need for early diagnosis of HIV infection, use of innovative approaches to reach and retain adolescents living with HIV in treatment and identifying specific groups, such as older adolescents, that are at high risk of dropping out of treatment for targeted care and support.

## Introduction

The burden of HIV in Africa is increasingly on adolescents and young adults. Worldwide, adolescents represent 41% of new HIV infections and are the only age group with increasing death rates due to AIDS [1]. Most of the adolescents living with HIV in sub-Saharan Africa (SSA) are girls and young women, who are particularly vulnerable due to such factors as early sexual debut, age disparate sexual partnerships, gender inequality and biological susceptibility [2]. Also of concern are adolescent members of key populations, including adolescents involved in sex work or using drugs, and young males who have sex with males [2].

Despite early successes in the HIV response in Uganda, HIV prevalence among the general population has steadily increased from 6.4% in 2005 to 7.3% in 2011. Risky sexual behaviours, inconsistent condom use, multiple sexual partnerships and low levels of male circumcision contribute to HIV acquisition and transmission in the country [3]. Like elsewhere in SSA, the HIV epidemic in Uganda continues to disproportionately affect young women [3,4]. Among adolescents aged 15 to 19 years, HIV prevalence is estimated at 2.4%, with a higher prevalence among females (3.0%) than males (1.7%) [4].

As antiretroviral therapy (ART) programmes are rolled out, the retention of adolescent patients in HIV care has gained more attention in recent years [5,6]. Yet adolescents face unique barriers to care and treatment, including being unaware of their sero-status due to a lack of disclosure, difficulties in transitioning from paediatric care to self-management, and family structural factors, in addition to the common psychosocial, economic, health systems and medical barriers faced by adult patients on ART [5,7]. Adolescents and young adults have significantly higher rates of loss to follow-up from HIV care and treatment than adults, which also contribute to their comparatively poorer outcomes [8,9].

Several strategies have been proposed for better engaging this population, including removing age-related barriers to care [10], developing new HIV testing modalities [11,12] and improving management of the transition from paediatric to adult care [13,14]. However, the evidence on retention and reducing loss to follow-up in HIV care programmes is limited for adolescents and targeted research is critical for improving treatment outcomes and reducing morbidity and mortality in this group 18 [6,15–18].

As one of the largest non-governmental ART programmes in Uganda, The AIDS Support Organization (TASO) was founded in 1987 with the aim of providing patient support for people living with HIV. In 2005, TASO started implementing a family-centred testing, treatment and counselling approach if there was a suspicion that any family member was HIV positive, after noticing that patients were sharing drugs with their family members, including parents sharing drugs with their children, and parents sharing drugs among themselves.

TASO's family-centred approach involved conducting home-based HIV counselling and testing to family members of the index patients. Those who tested positive within the family were assessed for ART eligibility using CD4 cell count and World Health Organization (WHO) staging, and if eligible, were linked to TASO's centre for treatment. Those not eligible were linked to TASO for appropriate care and support services and those who tested negative were counselled on risk reduction. TASO used the household-based approach in order to enhance access to testing, counselling and treatment services for all family members including their children and adolescents.

This paper examines the extent of retention in HIV care and the factors associated with attrition of adolescents aged 10 to 19 years in TASO's HIV treatment programmes in Uganda.

## Methods

### Study design

The study involved a retrospective secondary analysis of clinical data of adolescents aged 10–19 years collected from 10 of the 11 clinics operated by TASO. We excluded TASO Mulago because the site was not providing ART to adolescents during the study period (all adolescents from this site were linked for specialized ART care to Baylor Uganda). Data were extracted on HIV-positive adolescents from a central electronic database at TASO headquarters in Kampala.

### Study setting and data collection

As one of the largest non-governmental ART programmes in the country, TASO operates 11 ART service centres across all the regions of Uganda with funding from the United States President's Emergency Plan for AIDS Relief (PEPFAR). These centres are located in mainly rural parts of Uganda and serve a mean catchment area with a 75-km radius. As of 2015, the organization had provided treatment to over 68,020 clients, of whom more than 6% were children and adolescents.

TASO has implemented and revised several service delivery models over the past 10 years, including home-based care, satellite clinics, community drug distribution, as well as more conventional clinic-based approaches. Starting in late 2015, annual routine viral load monitoring is performed as part of TASO's ART programme. Adolescents who have been on ART for more than one year, and are aware of their HIV sero-status, are evaluated by clinicians and counsellors for downward referral to community drug distribution points (CDDPs). Community-drug distribution is a care model for stable patients designed to make ART delivery more efficient for the health system and provide appropriate support to encourage the long-term retention of patients. ART is provided at the community level by trained lay workers who are supervised by a clinical services supervisor. Adherence to drug regimens is evaluated by staff at the time of pill refill by asking patients to self-report the number of pills missed. Adolescents who have difficulty coping with decentralized ART care service at CDDPs are referred back up to the facility-based care delivery model.

Data were extracted from the pre-ART register, ART case evaluation forms, laboratory registry, death registry, ART commencement forms and drug refill forms. Each dataset contains a unique client identification number that merges information pertaining to the same individual from the different datasets. Information was collected on a total of 1228 HIV-positive adolescents aged 10–19 years who enrolled in ART between January 2006 and December 2011 from 10 TASO centres. Data extracted included clients’ socio-demographic characteristics, ART start date, treatment regimen, CD4 cell count at enrolment, WHO clinical staging and pharmacy refill data. The datasets also contained information on known deaths and patients who transferred out of the programme. Patient charts were used to supplement the information from the clinical datasets as necessary.

### Data analysis

The study outcomes of interest are HIV care retention and attrition. The indicators for retention; mortality, reported as death at TASO and attrition, were generated by identifying adolescents who enrolled in ART between 2006 and 2011 and had at least one clinic visit within the six months before 1 June 2013. Retention was defined as any patient who had at least one clinic visit in the six months before June 2013; was still alive at the end of June 2013, excluding those deaths reported to TASO stopped ART; or was lost to follow-up (LTFU) [19]. Attrition was defined as the number of adolescents whose deaths that were reported to TASO, who were LTFU or who stopped treatment by the end of June 2013. Patients were defined as LTFU if the last contact was more than three months before the end date of the observation period and they were censored at their last contact date with a TASO service. Married was defined as an adolescent who is married or co-habiting with a sexual partner during the review period.

Data were analyzed using descriptive statistics and multivariable regression models in Stata version 12 [20]. Multivariable Cox proportional hazards regression models was used to examine the factors associated with attrition. The results of the analysis are presented as adjusted hazard ratio (AHR) with 95% confidence intervals (CIs) and interpreted as the relative risk of attrition from ART programmes. The models adjusted for socio-demographic and clinical factors, which included age, sex, mode of ART delivery, CD4 at ART initiation and cohort year (i.e. year of initiation). The variables included in the model those found to be associated with retention in ART care in both the literature and TASO's experience, based on factors influencing retention in care among adolescents. A two-tailed statistical test with a *p*-value of <0.05 was considered to be statistically significant for all tests.

### Ethical approval

Patients’ records were anonymized and de-identified prior to analysis as per TASO's data protection and access policy. The study was approved by the Population Council Institutional Review Board and the Research and Ethics Committee of TASO, as well as registered with the Uganda National Council of Science and Technology (UNCST).

## Results

Data were collected on a total of 1228 adolescents who initiated ART between January 2006 and December 2011, with a median time on ART of four years (IQR=3–6 years). Over half (61%) were young adolescents (aged 10–14 years), 57% were females and 73% had a primary school education. At the time of ART initiation, 19% of patients had a CD4 count of <100/mm^3^ and 69% had a CD4 count of <250/mm^3^. Just over three-fifths (61%) obtained their drug refills from a health facility. A total of 792 (65%) adolescents received at least one clinical service in the six months preceding June 2013. For participants not retained in care, 36 (3%) were known to have died or to have transferred out, and 400 (32%) were classified as LTFU (Table 1).

**Table 1:** Baseline characteristics of adolescents aged 10–19 years in 10 TASO ART centres, 2006–2011 (*n*=1228)

Variable	*n*	Female	Male	Percentage of the totals (%)
Clinical outcome as of June 2013				
Alive and active in care	792	442	350	(65)
Dead	27	13	14	(2)
LTFU	400	236	164	(32)
Transferred	9	6	3	(1)
Site of participant				
Entebbe	103	58	45	(9)
Gulu	97	65	32	(8)
Jinja	99	53	46	(8)
Masaka	170	112	58	(14)
Mbale	163	92	71	(13)
Mbarara	131	71	60	(11)
Masindi	62	34	28	(5)
Rukungiri	131	68	63	(11)
Soroti	128	68	60	(11)
Tororo	144	76	68	(11)
Age (years)				
10–14	750	400	350	(61)
15–19	478	297	181	(39)
Highest level of education				
None	223	119	104	(18)
Primary	894	516	378	(73)
Secondary and above	111	62	49	(9)
Venue of ARV refill				
CDDP	476	278	198	(39)
Health facility	752	419	333	(61)
Marital status				
Single	1170	660	510	(78)
Married	58	37	21	(3)
CD4 at ART initiation				
<250 cells/mm^3^	851	486	365	(69)
≥250 cells/mm^3^	377	211	166	(31)
Year of ART initiation				
2006–2008	453	243	210	(37)
2009–2011	775	454	321	(63)
WHO stage (*n*=984)				
Stage 1&2	725	417	308	(74)
Stage 3	218	133	85	(22)
Stage 4	41	20	21	(4)

LTFU, lost to follow-up; CDDP, community drug distribution point.

In the bivariate analysis, the factors associated with attrition were TASO site (*p*<0.001), CD4 cell count at initiation (*p*<0.001), age of the adolescent at ART initiation (*p*<0.001), marital status (*p*=0.001) and year of ART initiation (*p*<0.001). TASO Masindi and Gulu facilities reported a higher attrition rate of adolescents than other centres (52 versus 38%, respectively). We also noted variations in the level of retention by site. In particular, the level of retention was lower at TASO Gulu (67%) and Masindi (52%), while TASO Jinja, Soroti and Rukungiri reported better retention as shown in Table 2.

**Table 2:** Characteristics of active (non-attrition) and non-active (attrition) adolescents aged 10–19 years in 10 TASO centres (*n*=1228)

	Active (*n*=792)				Non-active (*n*=436)				
			
	*n*	% of total sample	Male	Female	*n*	% of total sample	Male	Female	*P*
Site of participant									0.000
Entebbe	83	(8)	32	51	20	(10)	13	7	
Gulu	65	(6)	21	44	32	(16)	11	21	
Jinja	95	(9)	44	51	4	(2)	2	2	
Masaka	133	(13)	45	88	37	(18)	13	24	
Mbale	125	(12)	48	77	38	(19)	23	15	
Mbarara	119	(12)	55	64	12	(6)	5	7	
Masindi	32	(3)	18	14	30	(15)	10	20	
Rukungiri	126	(12)	61	65	5	(2)	2	3	
Soroti	127	(12)	60	67	1	(0)	0	1	
Tororo	121	(12)	55	66	23	(11)	13	10	
Age (years)									0.023
10–14	641	(62)	297	344	109	(54)	53	56	
15–19	385	(38)	142	243	93	(46)	39	54	
Highest level of education									0.005
None	193	(19)	86	107	30	(15)	18	12	
Primary	752	(73)	319	433	142	(60)	59	83	
Secondary and above	81	(9)	34	47	30	(15)	15	25	
Venue of ARV refill									0.670
CDDP	631	(65)	277	354	121	(60)	56	65	
Health facility	395	(35)	162	233	81	(40)	36	45	
Marital status									0.007
Single	983	(84)	426	557	165	(82)	8	7	
Married	43	(12)	13	30	10	(6)	84	103	
CD4 at ART initiation									
<250 cells/mm^3^	694	(68)	28	397	157	(78)	68	89	0.005
≥250 cells/mm^3^	332	(32)	142	190	45	(22)	24	21	
Year of ART initiation									
2006–2008	366	(36)	174	192	87	(43)	36	51	0.046
2009–2011	660	(64)	265	395	115	(57)	56	59	
WHO stage (*n*=984)	*n*=837				*n*=167				
Stage 1&2	626	(64)	259	367	118	(58)	49	50	0.161
Stage 3	177	(18)	69	108	42	(21)	16	25	
Stage 4	34	(3)	20	14	7	(3)	1	6	

In the multivariate Cox proportional hazards analysis, factors associated with attrition were age (AHR=1.29, 95% CI 1.01–1.65), CD4 at ART initiation (AHR=0.51, 95% CI 0.36–0.71) and site of participants: TASO Gulu (AHR=2.26; 95% CI 1.27–4.02), TASO Jinja (AHR=0.24, 95% CI 0.08–0.70) and TASO Masindi (AHR=3.30, 95% CI 1.87–5.84) (Table 3).

**Table 3:** Factors associated with attrition among adolescents aged 10–19 years in 10 TASO centres

	Univariate		Multivariate	
		
List of factors	HR (95% CI)	*p*	HR (95% CI)	*p*
Gender				
Female	Ref			
Male	1.03 (0.77–1.36)	0.858		
Site of participant				
Entebbe	Ref		Ref	
Gulu	2.02 (1.14–3.59)	0.016	2.17 (1.21–3.89)	0.009
Jinja	0.19 (0.07–0.56)	0.003	0.24 (0.08–0.71)	0.010
Masaka	1.32 (0.77–2.28)	0.314	1.37 (0.79–2.37)	0.266
Mbale	1.37 (0.80–2.36)	0.264	1.69 (0.97–2.98)	0.064
Mbarara	0.48 (0.23–0.98)	0.044	0.43 (0.21–0.91)	0.026
Masindi	2.98 (1.69–5.27)	0.000	3.50 (1.97–6.21)	0.000
Rukungiri	0.19 (0.07–0.51)	0.001	0.18 (0.07–0.49)	0.001
Soroti	0.04 (0.01–0.33)	0.002	0.04 (0.01–0.28)	0.001
Tororo	0.82 (0.45–1.50)	0.528	0.99 (0.54–1.82)	0.969
Age (years)				
10–14	Ref		Ref	
15–19	1.470 (1.11–1.94)	0.008	1.38 (1.02–1.86)	0.038
Highest level of education				
None	Ref		Ref	
Primary	1.21 (0.82–1.79)	0.342	0.91 (0.60–1.37)	0.644
Secondary and above	2.19 (1.31–3.66)	0.003	1.26 (0.72–2.19)	0.418
Venue of ARV refill				
CDDP	Ref			
Health facility	1.00 (0.76–1.34)	0.972		
Marital status				
Single	Ref		Ref	
Married	1.07 (0.70–1.63)	0.750	0.96 (0.62–1.49)	0.858
Minor	2.46 (1.19–5.12)	0.015	1.44 (0.68–3.06)	0.345
CD4 at ART initiation				
<250 cells/mm^3^	Ref		Ref	
≥250 cells/mm^3^	0.62 (0.44–0.87)	0.005	0.49 (0.34–0.69)	0.000
Year of ART initiation				
2006–2008	Ref		Ref	
2009–2011	1.92 (1.37–2.69)	0.000	2.11 (1.48–3.00)	0.000
WHO stage				
Stage 1 & 2	Ref			
Stage 3	1.25 (0.86–1.82)	0.228		
Stage 4	1.17 (0.54–2.52)	0.691		

**Figure 1:**
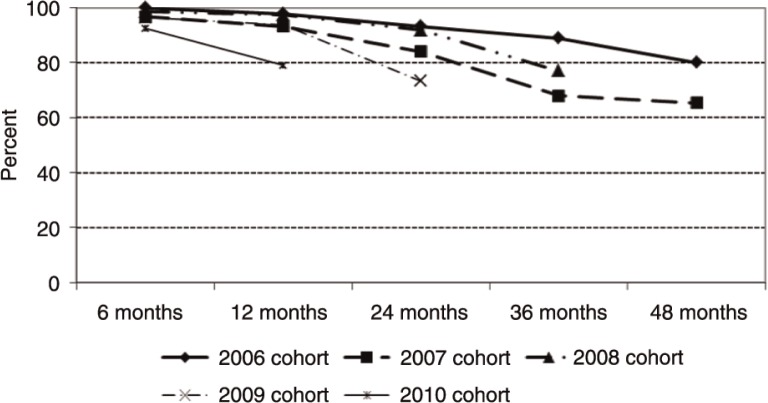
Trends in the level of retention and attrition of adolescents who initiated ART between 2006 and 2010 in 10 TASO centres.

Trend in retention analysis showed a higher hazard of attrition among adolescents that initiated ART between 2009 and 2010 (AHR=2.11, 95% CI: 1.48–3.00) compared to those that initiated ART between 2006 and 2008. Figure 1 shows that retention decreases over time for all cohorts of adolescents who initiated ART between 2006 and 2010; however, retention was consistently higher among the older cohort years (2006–2008) compared to the newer cohort years (2009–2010).

## Discussion

This study of 1228 adolescents in the TASO ART programme in Uganda demonstrated that nearly two-thirds (65%) of adolescents who initiated ART from January 2006 to December 2011 were retained in care. This finding is comparable to the five-year retention rate of 69% for adults in the same TASO programme [21]. Other studies in SSA have reported retention among adolescents as a major challenge for HIV programmes [3,22,23]. The long-term levels of retention seen in this study could be due to TASO's intensive resources targeting whole families with HIV testing, counselling, treatment and support services, especially in the earlier years of ART.

Retention and attrition rates, however, varied across the country's service centres. Treatment at the Gulu and Masindi centres was significantly associated with a higher risk of attrition. The lower retention rates seen in these centres could be due to their more rural location, the military conflict in the region during the study period, and the increased mobility of patients as part of post-conflict resettlement. The higher retention rates in TASO Soroti, Rukungiri, Jinja and Mbarara centres may be due to the presence of adolescent clinics that offer intensive pre-ART counselling to patients, including a follow-up home-based HIV counselling and testing visit to the family to identify an adherence support buddy. About one-third (32%) of adolescents were classified as LTFU in this study. It is possible that some adolescents may have died and their deaths were unreported, such that they were misclassified as LTFU. However, our finding is comparable to other studies that found LTFU among adolescents ranging from 17 to 30.3% at 24 months of follow up [24,25].

The risk of attrition was significantly lower among adolescents with a higher CD4 count compared to those with a lower CD4 count at the time of ART initiation. This finding is comparable to other research that reported adolescents with lower CD4 cell counts to be more likely to experience attrition compared to those with higher CD4 counts [26]. Additionally, the risk of attrition was not affected by patients’ clinical stage, which is not consistent with other studies reporting high rates of attrition among patients in advanced clinical stage disease [27,28]. This could be due to the psychosocial family support the patients received and the TASO home-based chronic care services delivered to patients who were in advanced clinical stages.

We found that the risk of attrition was significantly greater in older (15–19 years) than in younger (10–14 years) adolescents. This finding is comparable to other studies, one carried out in rural Zimbabwe and another in South Africa. The Zimbabwe study followed a cohort of adolescents who initiated ART between 2005 and 2008 [29]. The researchers found that older adolescents experienced greater LTFU than younger adolescents, with a rate per 100 person-years of 10.9 compared to 4.2. Retrospective data from seven South African clinics in urban Gauteng and rural Mpumalanga detected LTFU rates per 100 person years of 23.3 among older adolescents compared to 6.1 among younger adolescents [29,30]. The higher risk of attrition in older adolescents could be due to the challenges associated with transitioning from paediatric to adult care, given that the ages 15–19 years also mark an adolescent's transition to adulthood [8].

We also found that retention decreased among adolescents initiating ART in each successive year since 2006 and the gaps widened with longer durations of observation. This could be an indication of a change in TASO's programme from a family-centred approach to a clinic-based approach due to a decline in funding. Given that the 2015 WHO comprehensive ART guidelines removed CD4 and WHO clinical staging requirements for ART eligibility and the promotion of the “test and treat” strategy, more adolescents will be placed on ART, which could lead to strains on already resource-constrained health systems in Uganda and elsewhere in SSA. We, therefore, need to develop, pilot and fund innovative approaches for identifying and retaining HIV-positive adolescents in treatment programme if we are to achieve the UNAIDS 90-90-90 targets by 2020.

## Limitations

This study has several limitations. First, the type of data recorded in patients’ records is limited, preventing us from exploring further certain patterns in the data, such as the unexpected variations in the levels of retention of adolescents by the year of initiating ART or the risk of attrition between female and male adolescents. Second, data used in the analysis are routinely collected and entered by clinicians and might be subject to inaccuracies and incompleteness given the competing priority of ensuring the provision of quality services to clients. For instance, some variables like haemoglobin level, current education level, orphanhood and distance to an ART facility had extensive missing information and were not included in the multivariate analyses to avoid loss of statistical power. This could lead to under-estimation or over-estimation of the outcomes of interest. Third, the proportion of adolescents in TASO ART programmes that had died was based on health facility records; thus deaths that occurred at home might have been misclassified as LTFU.

## Conclusions

We found an overall retention rate of 65% among adolescents who initiated ART between 2006 and 2011, with varying durations in the ART programme. Retention was higher amongst adolescents who were younger (10–14 years of age), commenced ART in the early years of the study period and had higher CD4 counts at ART initiation. Advanced disease clinical stage at initiation was not associated with attrition, and retention varied across treatment sites.

The findings of this paper suggest that it is possible to achieve the long-term retention of adolescents in ART programmes. TASO's ART programme provides valuable lessons for improving the long-term uptake of treatment services by adolescents living with HIV. Retention in HIV care was highest when TASO's family HIV counselling approach was operating. This model enabled the TASO staff to conduct home-based HIV testing and counselling of the family members of clients, thus identifying children and adolescents living with HIV who could then be linked to HIV care. It also enhanced sero-status disclosure to the adolescents of their HIV status and of their parents, further facilitating retention in HIV care.

Variations in the risk of attrition from treatment sites and by clinical and socio-demographic characteristics suggest the need for early diagnosis of HIV infection, use of innovative approaches to reach and retain adolescents living with HIV on treatment, like TASO's family-centred approach, and identifying specific groups (such as older adolescents and female patients) that are at higher risk of dropping out of treatment for targeted care and support.
